# 932 Prolonged Midazolam Clearance and CYP3A4 Genetic Variants in a 4-year-old Pediatric Burn Patient

**DOI:** 10.1093/jbcr/iraf019.463

**Published:** 2025-04-01

**Authors:** Kristin Grimsrud, Tina Palmieri

**Affiliations:** University of California Davis; University of California Davis and Shriners Children’s

## Abstract

**Introduction:**

Variations in drug metabolism among patients pose a significant challenge in optimizing clinical care, especially in pediatric burn patients. Genetic variation is a key factor influencing drug metabolism, with cytochrome P450 (CYP) enzymes, particularly CYP3A4, playing a major role in metabolizing clinical drugs. Interestingly, midazolam can serve as a biomarker for CYP3A4 function, aiding in the evaluation of altered drug metabolism. The purpose of this abstract is to describe the prolonged elevation of midazolam levels in a child with burns compared to standard clinical doses in pediatric surgery and burn patients.

**Methods:**

Pediatric burn and surgery patients were enrolled in a study to evaluate fentanyl pharmacogenetics. On the day of surgery, blood samples were collected over a 10-hour period to assess drug concentrations and whole genome sequencing. Fentanyl and midazolam concentrations were measured using liquid chromatography-mass spectrometry, with midazolam serving as a biomarker for CYP3A4 function.

**Results:**

A total of 152 patients were enrolled, with the majority receiving midazolam before surgery. One notable case involved a 4-year-old 18 kg female burn patient, who was admitted the day prior to the burn unit intubated with 28% TBSA and suspected anoxic brain injury. She did not receive midazolam prior to surgery or at any time during her current hospitalization, yet midazolam was detected in her blood, with the maximum value exceeding 809 ng/ml, a potentially toxic dose. During her flight from Mexico, she received midazolam via continuous infusion at a rate of 600 µg/kg/hr for 4 hours, totaling approximately 43 mg. Genetic sequencing revealed that she carried two variants in CYP3A4 (*22/*36). In comparison, other patients in the study had midazolam levels ranging from 7.5-80.1 ng/mL in the first hour and 0.2-5.6 ng/mL 10-12 hours following a 2 mg intravenous midazolam administration.

**Conclusions:**

The midazolam detected in this patient likely originated from the administration during the previous day. The extremely high levels are attributed to the excessively high dose administered and the presence of the CYP3A4 *22/*36 variants, which likely impair metabolic function.

**Applicability of Research to Practice:**

These findings underscore the critical need for further investigation into the impact of genetic variants in clinical patients, particularly in pediatric burn patients. Additionally, this case highlights the need to re-evaluate current dosing practices for midazolam infusions, as well as other medications administered to critically ill patients, to prevent excessive dosing, especially when patients are nonresponsive and ventilated.

**Funding for the Study:**

Foundation Funding

